# Melatonin alleviates meiotic defects in fetal mouse oocytes induced by Di (2-ethylhexyl) phthalate *in vitro*

**DOI:** 10.18632/aging.101715

**Published:** 2018-12-26

**Authors:** Zhong-Yi Sun, Pan Zhang, Jun-Jie Wang, Jing-Cai Liu, Lan Li, Wei Shen, Qiu-Yue Zhai

**Affiliations:** 1 Center for Reproductive Medicine, Urology Department, Peking University Shenzhen Hospital, Shenzhen 518036, China; 2 Chengdu Women’s and Children’s Central Hospital, Chengdu 610031, China; 3 College of Life Sciences, Institute of Reproductive Sciences, Qingdao Agricultural University, Qingdao 266109, China; 4 School of Basic Medicine, Qingdao University, Qingdao 266071, China

**Keywords:** DEHP, melatonin, oocyte, meiosis, apoptosis

## Abstract

Di (2-ethylhexyl) phthalate (DEHP), an estrogen-like compound that is a ubiquitous environmental contaminant, has been reported to adversely affect human and mammalian reproduction. Many studies have found that exposure to DEHP during pregnancy perturbs female germ cell meiosis and is detrimental to oogenesis. Previous studies have demonstrated that melatonin (MLT) is beneficial to reproductive endocrinology, oogenesis, and embryonic development as the ability to antioxidative and antiapoptotic. However, whether the meiotic defect of germ cells exposed to DEHP could be rescued by MLT is not clear. Here, we cultured 12.5 days post coitum (dpc) fetal mouse ovaries for 6 days, exposed them to 100 μM DEHP with or without 1 μM MLT *in vitro.*. The results showed that DEHP exposure induced the abnormal formation of DNA double-strand breaks (DSBs), and inhibited the repair of DSBs during meiotic recombination. In addition, we found defective oocytes were prone to undergo apoptosis. Notably, this defect could be remarkably ameliorated by the addition of MLT via a reduction of the levels of reactive oxygen species and an inhibition of apoptosis. In conclusion, our data revealed that MLT had a protective action against the meiotic deterioration of fetal oocytes induced by DEHP in the mouse *in vitro.*

## Introduction

Mammalian reproduction is critical for perpetuating and diversifying the genetic information across generations [1,2], and normal germ cell development is critical for the genetic stability of a species [3]. In female, germ cells begin entry into meiosis during the fetal stage, which is fundamental to the production of viable gametes and the propagation of a sexually reproducing species [4]. During the first meiosis prophase (MPI), DNA double-strand breaks (DSBs) sever entire chromosomes and pose a severe hazard to genomic integrity because of chromosomal fragment loss or chromosomal rearrangements [5]. These DSBs can be repaired by homologous recombination (HR) dependent mechanisms, and ultimately establish links between unassociated homologs [6,7]. The regulated repair of meiotic DSBs by HR leads to the formation of crossovers which serve to maintain a strong connection between the homologous chromosomes, thus enabling the faithful segregation of homologs during MPI [8]. Notably, a recent study has reported that Di (2-ethylhexyl) phthalate (DEHP) exposure impairs MPI and DNA damage repair in female fetal mouse germ cells *in vitro*, and acts through multiple pathways including DNA damage and apoptosis pathways [9].

DEHP, a ubiquitous plasticizer with estrogen-like activity, is found extensively in common consumer goods including medical equipment, and building products [10]. Due to the characteristic of being noncovalently bound, it is readily leached from products and slips into the surrounding environment [11]. This widespread presence of DEHP leads to humans being exposed via multiple ways including oral ingestion, inhalation, and dermal contact [11-13]. DEHP and its metabolites have been detected in various human tissues such as blood, urine, amniotic fluid, umbilical cord blood, breast milk, and ovarian follicular fluid [14,15]. Several studies have demonstrated that DEHP acts as an endocrine-disrupting chemical (EDC) and has adverse effects on the female reproductive system via estrogen receptors (ERs) [16,17]. Robust evidences have shown that prenatal exposure to DEHP decreased litter sizes and body weight, disrupted sex determination, caused precocious puberty and reduced circulating estradiol levels in females [18,19]. In particular, DEHP exposure in pregnant mice impairs the offspring’s ovarian development including primordial follicle assembly, follicular development and oocyte maturation [20,21]. DEHP exposure also lead to increased levels of reactive oxygen species (ROS) during follicle growth which may be responsible for the poor oocyte quality observed [22]. Furthermore, the MPI of female fetal germ cells was delayed when either the fetus or isolated female germ cells were exposed to DEHP *in vitro* [9,23]. Moreover, germ cell cyst breakdown was also disturbed via the adverse effect of DEHP on gap junctions [9]. Alarmingly, exposure to DEHP altered the DNA methylation pattern of imprinted genes, suggesting that DEHP exposure may lead to heritable impairment of ovarian development [23].

The amine hormone melatonin (*N*- Acetyl- 5- methoxy tryptamine, MLT) is synthesized and released from the pineal gland and plays an important role in the female reproductive system [24,25]. As a multifunctional molecule, MLT and its derivatives exert various biological activities including having antioxidative and anti-apoptotic effects [26,27]. Additionally, it can directly scavenge ROS and upregulate the gene expression of several antioxidant enzymes [28,29]. Notably, MLT plays a role in regulating reproductive endocrinology, oogenesis, and embryonic development [30,31]. In fact, it has been found beneficial to embryonic development being partially attributed to its ability to decrease the expression of pro-apoptotic genes and increase the level of anti-apoptotic genes, as well as reduce ROS [30,32]. Interestingly, a recent study found that DEHP adversely affected prepuberal spermato-genesis and perturbed crucial epigenetic activities in male germ cells, and that MLT was able to prevent this damage [33]. In addition, Sun et al. found EDCs decreased oocyte quality and MLT improved oocyte maturation through its rescue effects on oocyte oxidative stress mediated apoptosis and autophagy [34].

Previous studies have reported the potential toxic effects of DEHP and the protective effects of MLT on the reproductive system, no reports have examined whether MLT can improve meiotic defect of fetal mouse oocytes induced by DEHP *in vitro*. Therefore, in this study, we focused on the effects of MLT on improving the meiotic defects of fetal mouse oocytes exposed to DEHP.

## RESULTS

### MLT relieves the abnormity of meiotic DSBs in DEHP-exposed fetal oocytes

**Figure 1:**
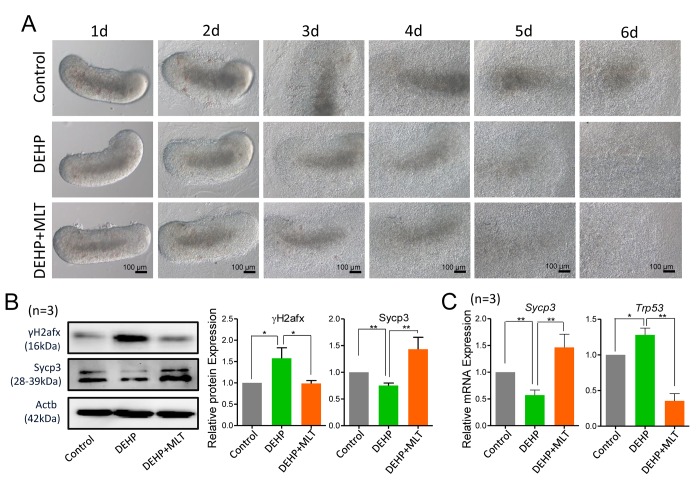
**Effects of MLT on meiotic progression and DSBs in DEHP-exposed fetal ovaries.** (**A**) Morphology of 12.5 dpc ovaries cultured for 6 days in control, DEHP and DEHP+MLT group *in vitro*. (**B**) Western blot analyses of the expression of Sycp3 and γH2afx protein in control, DEHP, and DEHP+MLT groups. (**C**) Relative expression level of genes *Sycp3* and *Trp53* in control, DEHP and DEHP+MLT groups. The results were presented as mean ± SEM. *P < 0.05, ** P < 0.01.

Previously, we found that DEHP exposure during gestation perturbs female germ cell meiosis and primordial follicle assembly [9,20]. To evaluate whether DEHP exposure induces meiotic defects in fetal oocytes, 12.5 dpc fetal mouse ovaries were cultured and exposed to 100 μM DEHP for 6 days*.* There were no morphologically alterations in ovaries exposed to DEHP and control groups (Figure 1A). However, the protein levels of the meiosis-related synaptonemal complex protein (Sycp3) and phosphorylated H2afx protein (γH2afx, marker of DSBs) were decreased and increased respectively in the DEHP treated group (Figure 1B; *P* < 0.05 or *P* < 0.01). DEHP exposure reduced the mRNA expressions of *Sycp3,* but markedly increased the mRNA levels of the DNA damage-related gene *Trp53* when compared with that of the control group (Figure 1C; *P* < 0.05 or *P* < 0.01).

To examine whether MLT can rescue the DEHP-induced meiotic defect, based on our previous study, we treated DEHP-exposed fetal mouse ovaries with a dose of 1 μM MLT [35]. Expectedly, MLT significantly increased the level of Sycp3 and reduced the level of γH2afx protein, consistent with the mRNA levels of *Sycp3* and *Trp53* compared with that of DEHP treatment group (Figures 1B and 1C; *P* < 0.05 or *P* < 0.01). Similarly, there were no morphological differences between the DEHP+MLT group and the DEHP group (Figure 1A).

**Figure 2:**
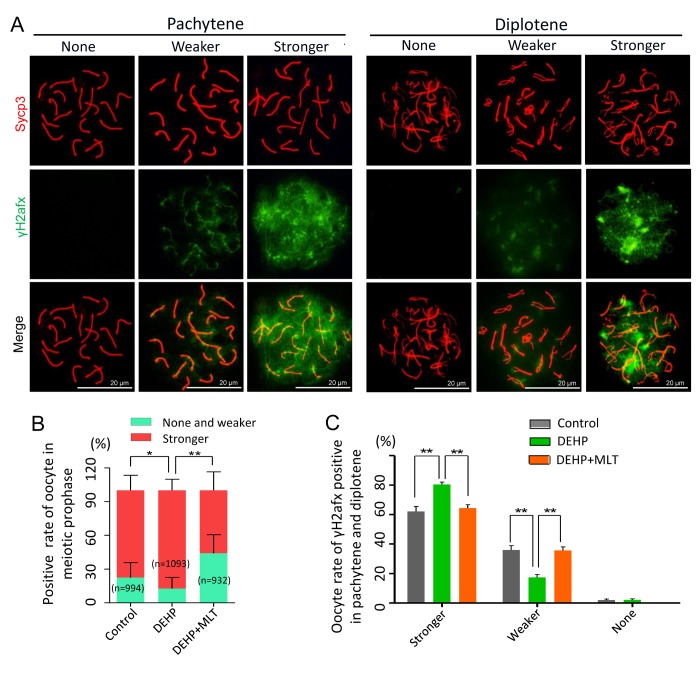
**The effect of MLT on the formation of DSBs in fetal oocytes.** (**A**)The immunofluorescence with Sycp3 (red) and γH2afx (green) in fetal oocytes. (**B**) The percentages of stronger, none and weaker γH2afx signal in oocyte in all MPI stages (control: 77.60 ± 5.04%, 22.40 ± 5.04%; DEHP: 87.24 ± 4.02%, 12.46 ± 4.02%; DEHP+MLT: 56.03 ± 9.52%, 43.97 ± 9.52%). (C) The percentages of stronger, weaker and none staining of γH2afx in pachytene and diplotene oocytes (control: 62.13 ± 3.37%, 35.92 ± 2.99%, 1.95 ± 0.78%; DEHP: 80.48 ± 1.53%, 17.41 ± 1.94%, 2.11 ± 0.78%; DEHP+MLT: 64.36 ± 2.39%, 35.64 ± 2.39%, 0.00 ± 0.00%). The results were presented as mean ± SEM. *P < 0.05, ** P < 0.01.

To further prove the reliability of the results, we double stained oocyte cytospreads for Sycp3 and γH2afx (Figure 2A). The expression pattern of γH2afx in oocytes could be classified into three main categories: none (negative or rare barely detectable foci), weaker (a few patches and less than 50% area) and stronger (numerous small and large patches, more than 50% area) [36,37]. Statistical analysis at all MPI stages indicated that the percentage of oocytes with a stronger staining pattern of γH2afx significantly increased in the DEHP treated group (87.24 ± 4.02%) compared with that of the control group (77.60 ± 5.04%) (*P* < 0.05), but that was much lower in the DEHP+MLT treated group (56.03 ± 9.52%) (Figure 2B; *P* < 0.01). Since most oocytes were in the pachytene and diplotene at 18.5 dpc, for purpose of studying the expression of γH2afx in oocytes, we further separately classified them into three categories. Similarly, the percentage of stronger γH2afx oocyte in DEHP + MLT group (64.36 ± 2.39%) is closer to control group (62.13 ± 3.37%), and much higher in DEHP group (80.48 ± 1.53%) (Figure 2C; *P* < 0.01).

### MLT restores the ability of DSBs repair induced by DEHP in fetal oocytes

**Figure 3:**
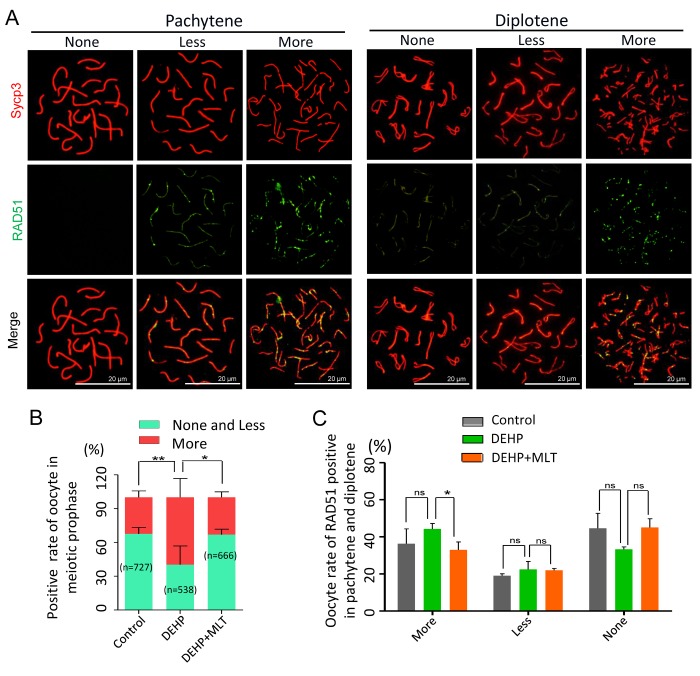
**Effects of MLT on HR in DEHP-exposed fetal oocytes.** (**A**)The immunofluorescence with Sycp3 (red) and RAD51 (green) in fetal oocytes. (**B**) The percentages of more, less and none staining of RAD51 in all MPI stages (control: 32.48 ± 2.54%, 67.52 ± 2.54%; DEHP: 59.83 ± 7.44%, 40.17 ± 7.44%; DEHP+MLT: 33.13 ± 2.79%, 66.87 ± 2.79%). (**C**) The percentages of more, less and none staining of RAD51 in pachytene and diplotene oocytes (control: 36.27 ± 8.02%, 19.09 ± 1.03%, 44.64 ± 8.08%; DEHP: 44.24 ± 2.98%, 22.50 ± 4.28%, 33.26 ± 1.30%; DEHP+MLT: 32.97 ± 4.27%, 22.00 ± 1.03%, 45.03 ± 4.76%). The results were presented as mean ± SEM. *P < 0.05, ** P < 0.01.

As the repair of meiotic DSBs is crucial for HR and crossover formation, we then performed chromosome staining for the proteins RAD51 and Sycp3 (Figure 3A). The RAD51 staining pattern of meiotic oocytes was classified into three categories: none (negative), less (numbers of RAD51-positive foci ≤ 10 in each oocyte) and more (numbers of RAD51-positive foci > 10 in each oocyte) according to previous studies [38,39]. The statistical results at all MPI stages showed the percentage of positive-cells with more RAD51 signal in the DEHP group (59.83 ± 7.44%) was significantly higher than that of the control group (32.48 ± 2.54%) (Figure 3B; *P* < 0.01). Meanwhile, compared with the DEHP treated group, the proportions of RAD51 positive-cells in the DEHP+MLT group (33.13 ± 2.79%) was decreased (Figure 3B; *P* < 0.05). Likewise, there were not signiﬁcant differences between the control (36.27 ± 8.02%) and DEHP + MLT (32.97 ± 4.27%) group in terms of the percentage of positive oocytes with more RAD51 signal at pachytene and diplotene stage, but the rate was increased in DEHP group (44.24 ± 2.98%) (Figure 3C).

### MLT improves the defect of mismatch repair caused by DEHP in fetal oocytes

**Figure 4:**
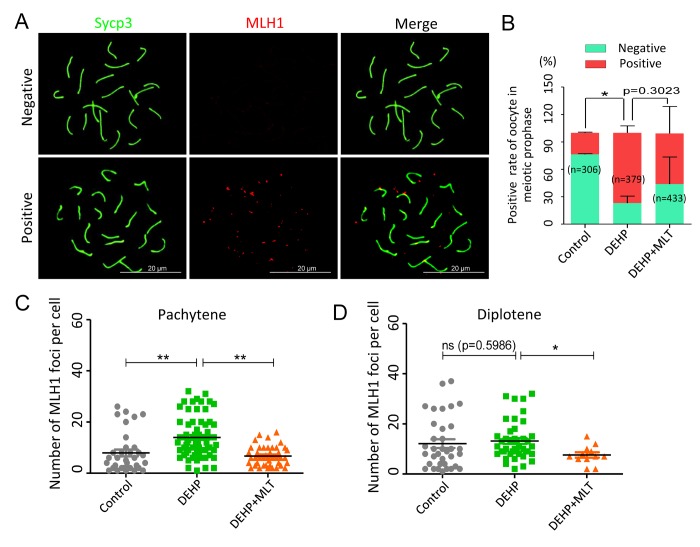
**Effects of MLT on mismatch repair in DEHP-exposed fetal oocytes.** (**A**)The immunofluorescence with Sycp3 (green) and MLH1 (red) in fetal oocytes. (**B**) The percentages of positive and negative MLH1 signal in oocytes (control: 23.63 ± 0.55%, 76.37 ± 0.55%; DEHP: 77.01 ± 4.41%, 22.99 ± 4.41%; DEHP+MLT: 55.04 ± 17.04%, 43.87 ± 17.08%). (**C** and **D**) The amounts of the MLH1 positive foci in pachytene (control: 7.91 ± 1.33; DEHP: 13.97 ± 0.95; DEHP+MLT: 6.74 ± 0.58) and diplotene (control: 12.11 ± 1.74; DEHP: 13.18 ± 1.16; DEHP+MLT: 7.58 ± 1.07) oocytes, respectively. The results were presented as mean ± SEM. *P < 0.05, ** P < 0.01.

Generally, successful mismatch repair is a conserved DNA repair pathway and plays a crucial role in meiotic crossover and DNA recombination. MLH1 facilitates both mismatch repair and crossover during meiosis [40]. To verify the effect of MLT on mismatch repair in the germ cells following DEHP exposure, MLH1 staining was carried out to identify the sites of exchange (Figure 4A). Based on the results of the statistical analysis, DEHP exposure significantly increased the rate of MLH1 positive oocytes (77.01 ± 4.41% vs 23.63 ± 0.55%, *P* < 0.05), and this increase had no significant improvement when MLT was added with DEHP (55.04 ± 17.04%) (Figure 4B). The average number of MLH1 foci in per cell showed the MLH1 foci number of oocytes at pachytene with DEHP exposure dramatically increased (13.97 ± 0.95) when compared to the control group (7.91 ± 1.33) and deceased in the presence of MLT (6.74 ± 0.58) (Figure 4C; *P* < 0.01). However, after MLT-administrated, the average number of MLH1 foci in per cell at diplotene (7.58 ± 1.07) was significantly decreased when compared to the DEHP treated group (13.18 ± 1.16) (*P* < 0.05), although there were not significant changed in control group (12.11 ± 1.74) compared to the DEHP group (Figure 4D). Collectively, these data indicated that DEHP exposure of fetal oocytes would affect the expression of MLH1, which might be one of the critical factors leading to the failure of mismatch repair. As expected, MLT alleviates mismatch repair abnormalities by influencing the level of MLH1.

### MLT decrease ROS levels and suppresses apoptosis in DEHP-exposed fetal ovaries

**Figure 5:**
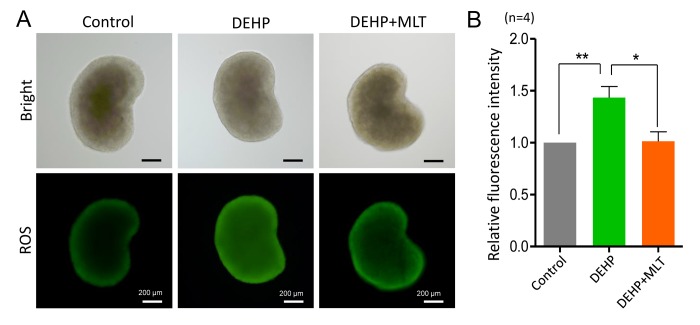
**Effects of MLT on ROS generation in the fetal ovaries exposure to DEHP.** (**A**) Representative images of DCHF-DA fluorescence (green) in the ovaries from the control, DEHP and DEHP+MLT groups. (**B**) Fluorescence intensity of ROS level. The results were presented as mean ± SEM. *P < 0.05, ** P < 0.01.

Based on previous studies [34,41], we hypothesized that DEHP exposure would induce oxidative stress which would be accompanied by apoptosis in fetal oocytes. This would lead to the deterioration of critical regulators and events during oocyte meiosis. To confirm this assumption, we assessed the level of ROS in the control, DEHP, and DEHP+MLT groups (Figure 5A). In the DEHP treatment group, the fluorescent intensity of ROS was significantly increased compared to that of the control group (*P* < 0.01), while following the administration of MLT, ROS generation reduced dramatically (Figure 5B; *P* < 0.05).

**Figure 6:**
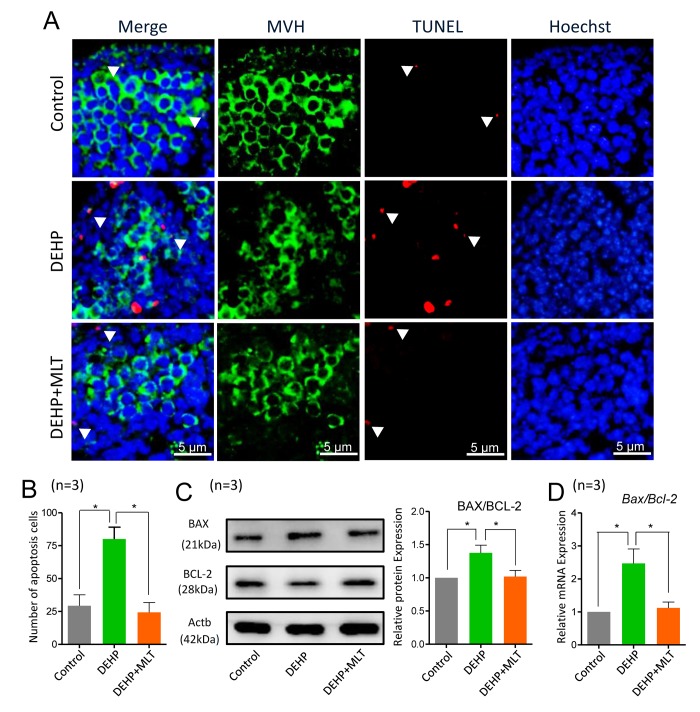
**Effects of MLT on apoptosis in DEHP-exposed fetal ovaries.** (**A**) Representative images of TUNEL-staining in control, DEHP, and DEHP+MLT groups. (**B**) The number of apoptosis positive cells in the different groups. (**C**) The protein expression of BAX and BCL-2 by western blot in the control, DEHP and DEHP+MLT groups. (**D**) The relative expression of *Bax and Bcl-2* genes in the control, DEHP and DEHP+MLT groups. The results were presented as mean ± SEM. *P < 0.05.

Next, we detected the apoptotic signals by TUNEL-staining in different groups. The results showed that apoptosis was hardly detected in control ovaries, but clearly existed in the DEHP-exposed group (Figure 6A). The number of TUNEL-positive cells was dramatically higher in the DEHP group when compared with that of the control group (*P* < 0.05) but was reduced in the DEHP+MLT group (Figure 6B; *P* < 0.05). Additionally, we further evaluated whether MLT influenced the expression levels of apoptotic proteins and genes, including BAX and the anti-apoptotic protein BCL-2. As expected, the ratios of BAX/BCL-2 proteins were significantly increased in the DEHP treated group compared with that of the control group (Figure 6C, *P* < 0.05), however, the administration of MLT restored the protein levels (*P* < 0.05). This observation was consistent with the gene levels (Figure 6D; *P* < 0.05).

## DISCUSSION

There is ample evidence showing that DEHP adversely impacts the mammalian reproductive system [15,42] when exposure occurs during pregnancy. As DEHP impairs fetal ovary development by impairing oocyte meiosis, this study was conducted to determine whether the meiotic impairment of female fetal germ cells induced by DEHP could be improved by MLT. In the present study, we clarified the toxic effects and possible mechanisms of DEHP exposure on fetal oocyte meiosis and confirmed the action of MLT on ameliorating DEHP-induced meiosis defects. To test it, we examined the MPI, DSBs and the ability to repair DSBs on fetal oocytes following DEHP exposure in mice. DSBs sever entire chromosomes and pose a major threat to genome integrity with unpaired DSBs leading to cell death. In this study, the results demonstrated that MLT treatment decreased the number of γH2afx-positive cells in ovaries when compared with DEHP treated. This suggests MLT plays a key role in restoring DSB induced by DEHP. Moreover, HR and crossover during MPI are required for normal meiosis in mice, and its absence leads to abnormal meiotic division and sterility in both sexes. Our finding further showed that both the expressions of RAD51 and MLH1 were lower in oocytes from ovaries treated with MLT when compared to DEHP treated. This suggests that MLT could, at least partially, restore the defects related to DSBs repair caused by DEHP exposure. Collectively, in agreement with previous studies [9], DEHP exposure inhibited the MPI progression of fetal oocytes via inducing DSBs and damaging the ability to repair DSBs. As expected, MLT treatment substantially restored DSB repair, suggesting that MLT indeed has the potential to improve the quality of fetal oocytes exposed to DEHP.

We then explored the underlying mechanisms resulting in improvement from MLT in DEHP treated fetal ovaries. Increasing studies have shown that mammalian gametes and embryos are particularly vulnerable to oxidative stress due to their plasma membrane composition [43,44]. In the present study, we observed higher oxidative stress in DEHP-exposed fetal mouse ovaries, and lower in the MLT-treated ones compared to untreated controls. Importantly, the production of ROS is accompanied with apoptosis triggering ovarian somatic cell apoptosis and leading to apoptosis of the supported oocytes [45]. Our study is consistent with previous reports that DEHP exposure induces apoptosis in fetal ovaries [9], we also showed MLT administration rescued the toxic effects of DEHP on cellular apoptosis in exposed fetal ovaries. Thus, we believe MLT as a powerful antioxidant and anti-apoptotic agent which can scavenge toxic oxygen derivatives and reduce the formation of ROS and further prevent apoptosis in fetal ovaries. Moreover, the participation of H3K9me2 induced with MLT treatment seemed to be indispensable for the rescue activity of DEHP damage [33]. In mammalians, it has been established that DNA methylation and histone modification dynamics is vital during embryonic development. As reported, DEHP exposure could affect the DNA methylation of imprinting genes in fetal mouse germ cells [46]. Also, in the present research, the meiotic DSBs and recombination of oocyte with DEHP exposure were impaired, which implied the alteration of epigenetic modification with MLT might be another potential mechanism for the rescue, it still needs further confirmation.

Interestingly, MLT and the enzymes required for its synthesis were discovered in the human placenta [47]. What’s more, studies have shown that MLT is rapidly transferred from the mother to the fetus through the placenta, and the current opinion supports that fetal MLT levels are similar to those of the mother [48-50]. In fact, MLT in the placenta is potentially associated with preeclampsia, during severe preeclampsia, placental MLT levels as well as its receptors are depressed [51]. Preeclampsia is a serious disorder with ROS production being elevated [44], thus, as a potent antioxidant MLT may be beneficial to treat preeclampsia. These findings suggest MLT may be helpful in protecting fetal oogenesis in pregnant mothers exposed to DEHP.

## Conclusion

In conclusion, our results indicate that DEHP is toxic to the meiotic progress of fetal oocytes, showing increased DSBs formation and decreased DSBs repair. We further investigated oxidative stress and apoptosis as potential mechanisms for defects in fetal ovaries exposed to DEHP, and MLT is a promising agent for preventing DEHP induced toxicity.

## MATERIALS AND METHODS

### Animal bread and ethics statement

All animals used in this study were CD-1 mice purchased from Qingdao Dacheng Furen Co. LTD (Qingdao, China). Mice were housed in temperature and light controlled environments (21 - 22 °C; 12 hours light/12 hours dark cycle). Females were mated with males at 17:00 pm and the presence of a vaginal plug was checked the next morning at 8:00 am, when detected the day was considered 0.5 dpc. All procedures described in this study were reviewed and approved by the Ethical Committee of Shenzhen Hospital of Peking University.

### Ovarian culture

12.5 dpc fetal mouse ovaries were isolated and cultured as previously described [52]. Briefly, fetal mouse ovaries were dissected in half from 12.5 dpc embryos and cultured in 600 µL culture medium, consisting of α-minimal essential medium (α-MEM; Hyclone, SH30265.01B, Beijing, China), 10% fetal bovine serum (FBS; Gibco, 10099-141, USA), 0.23 mM sodium pyruvate (Hyclone, SH40003-12), 100 IU/ml of penicillin G, and 100 mg/ml of streptomycin sulfate, 10 mIU/ml follicle stimulating hormone (FSH; RD, 5925-FS, USA). Ovaries were cultured in humidified incubator at 37 °C, 5% CO_2_ in air and medium was changed every other day. At day six, the ovaries were collected and kept for further analysis. *In vitro* cultured ovaries were exposed to DEHP at a dose of 100 μM and 1 μM MLT, 0.1% DMSO and 0.1% alcohol were added to the culture as a vehicle control.

DEHP was obtained from Sigma-Aldrich (36735-1G, Saint Louis, MO, USA). Based on our previous study [9], DEHP was prepared at the concentrations of 0.254 M and 2.54 M in dimethylsulfoxide (DMSO) and the final concentration in the culture medium was 100 μM. Melatonin (M5250-1G) was purchased from Sigma-Aldrich and dissolved in 100% ethanol, which was added to the culture medium at the final concentration of 1 μM.

### Oocyte cytospreads

Staining of meiotic chromosomes were evaluated using oocyte cytospreads as previous study [53]. Ovaries were collected and incubated in hypo extraction buffer for 1.5 hours, fixed with 1% PFA overnight at room temperature. Fixed samples were blocked with antibody dilution buffer (ADB) at 37 °C for half an hour and incubated with primary antibodies (Table S1) at 37 °C for 8 hours. The slides were further blocked with ADB overnight at 4 °C. The next day, the slides were incubated with secondary antibodies of CY3 (anti-rabbit, Beyotime, A0516, Nantong, China; anti-mouse, Beyotime, A0568) or FITC- labeled goat IgG (anti-mouse, Beyotime, A0521; anti-rabbit, Beyotime, A0516) at 37 °C for 1.5 hours in the dark. Hoechst 33342 (Beyotime, C1022) was used to stain nuclei for 5 min and slides were mounted with Vectashield (Vector, H-1000, Shanghai, China). Finally, slides were analyzed under a fluorescence microscope (Olympus BX51, Japan). Experiments were repeated at least 3 to 4 times and every independent experiment for oocyte counting was at least 250.

According to the characteristic morphologies of chromosomes after cytospread staining with a Sycp3 antibody, the meiotic progression was clarified into leptotene, zygotene, pachytene, and diplotene. In the leptotene, chromosomes are decondensed and long. At zygotene, chromosomes become increasingly longer and formed the synaptonemal complex (SC). During pachytene, chromosomes are short and condensed, and homologs are fully synapsis. Entering diplotene, chiasmata are formed during homologous and the SC starts to disassemble [54].

### ROS level assay

To determine the levels of intracellular ROS production, fetal mouse ovaries were collected after culturing for 6 days *in vitro* and were then washed three times with phosphate buffer saline (PBS). Next, ovaries were incubated with the oxidation-sensitive florescent probe dichlorodihydrofluorescein (DCFH) for 30 min at 37 °C in D-PBS that contained 10 μM DCFH diacetate (DCFH-DA) (Beyotime, E004). After washing three times in D-PBS containing 0.1% BSA, ovaries were placed on glass slides, and the florescence intensity of each ovary was measured with a fluorescent microscope (Olympus BX51, Japan). For fluorescence intensity, the photographs of each independent experiment were taken under microscope with the same parameter settings (exposure time and intensity). The quantify of fluorescence intensity was examined with the ImageJ software, with the option of Plugins, the RGB measure of Analyze item was applied, the green channel then represented the fluorescence intensity of ROS.

### TUNEL staining

Bright Red Apoptosis Detect Kit (Vazyme, A113-02, China) was used for the detection of TUNEL positive cells as previously described [55]. Briefly, paraffin sections were heated at 60 °C for 2 hours, washed in xylene and rehydrated through a series of ethanol gradations. After antigen retrieval at 96 °C, sections were blocked and incubated with anti-MVH protein at 4 °C overnight, then FITC-labeled goat anti-rabbit IgG was used as a secondary antibody. Washed three times before sections were treated with proteinase K for 10 min, then according to the manufactures’ instructions, carried on an incubation with the TUNEL treatment mixture at 37 °C for an hour. Finally, nuclei were counterstained with Hoechst 33342. Images were taken under a fluorescence microscope (Olympus, BX51).

### Western blot

Proteins were extracts from ovaries using Cell Lysis Buffer (Beyotime, P0013) for western blot analysis according to standard methods [56]. The proteins were separated on a 10% SDS-PAGE gel and transferred onto polyvinylidene fluoride membrane (PVDF, immobilon-P^SQ^ transfer membranes, Millipore, ISEQ00010, USA). The membrane was blocked with TBST buffer (tris buffered saline, with tween-20) containing 5 - 10% BSA and incubated in primary antibody (Table S1) overnight at 4 °C. After three washes in TBST, the membrane was incubated with HRP conjugated goat anti-rabbit or anti-mouse IgG (Beyotime, A0216) diluted in TBST at room temperature for 1.5 hours. Finally, the membranes were reacted with BeyoECL Plus Kit (Beyotime, P0018). IPWIN software was used for density measurements.

### RNA extraction and quantitative RT-PCR

Total RNA was extracted from ovaries using the RNA prep pure Micro Kit (Aidlab, RN28, Beijing, China) according to the manufacturer’s instructions. Then, the RNA was reverse-transcribed into cDNA using TransScript One-Step gDNA Removal and cDNA Synthesis SuperMix (TransGen Biotech AT311-03, Beijing, China). All primers used in this research are listed in Supplementary Table S2. Relative quantification analysis was carried out with a LightCycler 480 II (Roche) using SYBR^®^ Premix Ex Taq™ II (TAKARA, RR820A, Dalian, China). Gene expression levels were calculated using β-actb for normalization. Relative transcript abundance was calculated using the 2^-ΔΔCt^ method [57].

### Statistical analysis

Results were obtained from at least three independent experiments and are expressed as mean ± SEM. Data were statistically analyzed with GraphPad Prism software and significant difference was determined with a Student’s unpaired *t-*Test of independent samples. *P* < 0.05 was considered as significant difference, while *P <* 0.01 was a highly significant difference.

## Supplementary Material

Supplementary Tables
