# Supplementary material for: Melatonin alleviates meiotic defects in fetal mouse oocytes induced by Di (2-ethylhexyl) phthalate in vitro
Source: Aging (Albany NY). 2018 Dec 26;10(12):4175–87. doi: 10.18632/aging.101715 (PMC6326675; doi:10.18632/aging.101715)
Supplement: Supplementary Tables [file aging-10-101715-s001.pdf]

SUPPLEMENTARY TABLES

Table S1. Primary antibodies.

Antibody	Vendor; Cat. No.	Dilution	Applications
Sycp3(Rb)	Novus Biologicals; NB300-232	1:1000	Western Blot
		1:200	Immunofluorescence
Sycp3(Mus)	Abcam; ab97672	1:200	Immunofluorescence
γH2afx	Abcam, ab26350	1:1000	Western Blot
		1:200	Immunofluorescence
RAD51	Abcam; ab133534	1:200	Immunofluorescence
MLH1	BD Pharmingen, 551091	1:200	Immunofluorescence
MVH	Abcam, ab13840	1:200	Immunofluorescence
BAX	Cell signaling, #2772s	1:1000	Western Blot
BCL-2	Beyotime, AB112	1:1000	Western Blot
β-Actb	Sangon Biotech; D110001	1:1000	Western Blot

Table S2. Primers used for quantitative RT-PCR.

Genes	Genbank	Forward primer sequence	Reverse primer sequence	Product Length (bp)
Actin	NM_007393.3	TCGTGGGCCGCTCTAGGCAC	TGGCCTTAGGGTTCAGGGGGG	255
Trp53	NM_001127233	ACAGTCGGATATCAGCCTCG	GCTTCACTTGGGCCTTCAAA	159
Sycp3	NM_011517.2	GGGGCCGGACTGTATTTACT	AGGCTGATCAACCAAAGGTG	169
Bax	NM_007527	ATGCGTCCAAGGAAGACTGAG	CCCCAGTTGAAGTTGCCATCAG	162
Bcl-2	NM_009741.5	GCAGAGATGTCCAGTCAG	CACCGAACTCAAAGAAGG	127
